# Infectious bacteria as biological warfare agents: mechanisms, epidemiological threats, and defense strategies

**DOI:** 10.3389/fbioe.2026.1805294

**Published:** 2026-05-18

**Authors:** Kamran Shah, Muhammad Imran Khan, Yanbing Guo, Muhammad Adnan, Hongzhi Wu

**Affiliations:** 1 College of Landscape and Horticulture, Yunnan Agricultural University, Kunming, Yunnan, China; 2 State Key Laboratory for Development and Utilization of Forest Food Resources, Zhejiang A&F University, Hangzhou, China; 3 Zhejiang Key Laboratory of Non-wood Forest Products Quality Regulation and Processing Utilization, Zhejiang A&F University, Hangzhou, China; 4 College of Veterinary Sciences, Faculty of Animal Husbandry and Veterinary Sciences, The University of Agriculture, Peshawar, Pakistan

**Keywords:** aerosol transmission, bacterial bioweapons, biodefense strategies, immune evasion, toxin-mediated pathogenesis

## Abstract

Infectious bacteria remain among the most plausible agents for deliberate biological attack because they combine environmental robustness, low infectious dose, and the capacity for rapid, severe disease. This review synthesizes current knowledge on major bacterial biowarfare threats including *Bacillus anthracis*, *Yersinia pestis*, *Francisella tularensis*, *Brucella* spp., and *Clostridium botulinum* across three levels: transmission routes, molecular pathogenesis, and defense strategies. We summarize how aerosol, food-water, and vector-borne pathways, together with globalization and urban crowding, shape outbreak potential, and contrast non-contagious threats such as inhalational anthrax with highly transmissible pneumonic plague. At the mechanistic level, we highlight convergent virulence platforms capsules and stealth surfaces, intracellular survival programs, type III/VI secretion systems, and toxins such as anthrax lethal toxin and botulinum neurotoxin that delay immune recognition and compress the window for effective intervention. We then review advances in surveillance and countermeasures, including portable PCR and CRISPR-based diagnostics, next-generation anthrax vaccines, antibiotics and antitoxins for plague and botulism, and emerging decontamination technologies for persistent spores. Finally, we discuss the integration of these tools within CBRNE incident management and global biosecurity frameworks, emphasizing persistent gaps in environmental remediation, resistance surveillance, and capacity in low-resource settings. Together, these data define priorities for strengthening resilience to bacterial biowarfare and bioterrorism.

## Graphical Abstract

**Figure:**
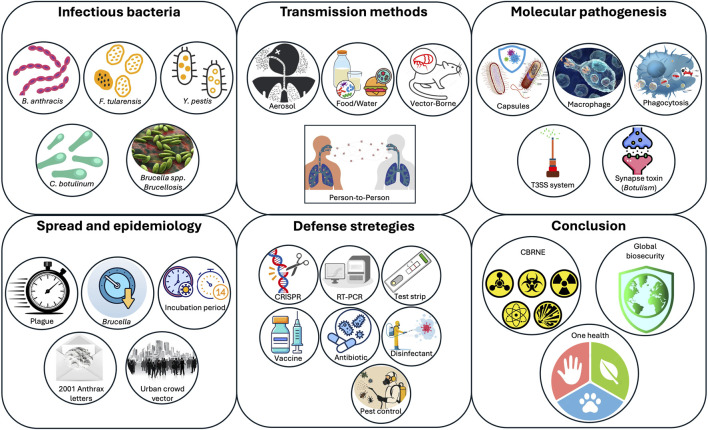

## Highlights

- *B. anthracis*, *Y. pestis*, *F. tularensis* are high priority biothreats due to high lethality and persistence.
- These bacteria evade immunity through toxins, immune suppression, and intracellular survival.
- Aerosol, contaminated food/water, and vector-borne routes are primary spread mechanisms.
- Rapid diagnostics, vaccines, and antibiotics improve preparedness, but global coordination is crucial for effective response.
- Persistent spores, like anthrax, present significant environmental cleanup challenges.

## 1 Introduction

The deliberate use of infectious bacteria as agents of biological warfare (BW) represents an ongoing and evolving threat to global security, public health, and geopolitical stability. BW is defined as the intentional deployment of pathogenic agent explicitly including bacteria and viruses, which have been weaponized historically to harm humans, livestock, or agricultural systems (Riedel, 2004). While viruses are historically significant to the biological weapons threat landscape, this review focuses specifically on infectious bacteria. This tactic exploits the virulence, environmental resilience, and transmissibility of pathogens to achieve strategic military or terrorist objectives. Historical accounts of BW are often debated. The most plausible instance from the pre-modern era is the medieval siege of Caffa (1346), where Mongol forces are reported to have catapulted *Yersinia pestis*-infected cadavers into the city (Wheelis, 2002). However, this is not a clear-cut act of BW. The account relies on a single source, the intent of the Mongols is unknown, and the method is epidemiologically questionable as plague is primarily flea-borne and not efficiently spread via corpses (Harris, 1995; Seth Carus, 2017). Despite this ambiguous precedent, the 20th century saw the rise of state-sponsored programs, most notably Japan’s WWII-era program (Unit 731), which weaponized a variety of pathogens, with *Yersinia pestis* (plague) being a primary and notably deployed agent alongside *Bacillus anthracis* (Manchee et al., 1981; Olson, 1999).

Beyond state-level programs, the enduring environmental threat of bacterial agents was starkly demonstrated by the contamination of Gruinard Island, Scotland, during WWII Allied bioweapons testing (1942–1943). Intentional release of *B. anthracis* spores rendered the island uninhabitable for decades, necessitating an extensive decontamination effort (formaldehyde and seawater) only declared complete in 1990. This episode serves as a potent lesson in the extreme persistence of anthrax spores and the long-term ecological disruption possible with even localized BW deployment (Manchee et al., 1981). Decades later, the 1990s attempts by the non-state Aum Shinrikyo cult in Japan highlighted the feasibility and challenges of BW acquisition and deployment by terrorist groups. Despite significant resources and scientific expertise, their multiple attempts to disperse *B. anthracis* spores and botulinum toxin in Tokyo (1990–1993) largely failed to cause mass casualties, primarily due to difficulties in achieving effective aerosolization and using less virulent strains. Nevertheless, these attempts underscored the evolving threat of bioterrorism by non-state actors possessing dual-use biotechnology capabilities and reinforced the critical importance of robust detection and interdiction measures (Olson, 1999; Takahashi et al., 2004). Such historical precedents underscore the multi-use potential of microbiological research, where scientific advancements can be perverted for malevolent purposes (Tucker, 2012).

The Cold War era further intensified bioweapon development, epitomized by the accidental release of *B. anthracis* spores in Sverdlovsk, USSR (1979), which caused numerous fatalities and demonstrated the catastrophic consequences of aerosolized bacterial agents (Meselson et al., 1994). In the 21st century, bacterial agents continue to pose central bioterrorism threats, exemplified by the 2001 U.S. anthrax attacks (Jernigan et al., 2001). These events starkly demonstrated the threat posed by bacterial pathogens that are categorized as Category A priority agents by the Centers for Disease Control and Prevention (CDC), a classification that predates the 2001 attacks and includes *B. anthracis*, *Y. pestis* (plague), and *F. tularensis* (tularemia) (Rotz et al., 2002). Their low infectious doses, high mortality rates, and potential for airborne transmission render them particularly suited for covert attacks (Inglesby et al., 2002). Key characteristics of these and other bacterial agents prioritized by the CDC for biodefense preparedness are summarized in Table 1.

**TABLE 1:** Classification of key bacterial biowarfare agents according to CDC categories.

CDC category	Pathogen (disease)	Primary mode of transmission	Severity of disease (untreated case-fatality rate)	Potential for use as BW agent
A (Highest risk: high mortality, easy dissemination, public panic potential)	*Bacillus anthracis* (Anthrax)	Aerosol (inhalation), cutaneous contact, gastrointestinal	∼100% (inhalation); 20%–80% (cutaneous)	High: Spore persistence enables covert aerosol release; historical use (e.g., 2001 U.S. attacks); engineerable for antibiotic resistance
	*Yersinia pestis* (Plague)	Aerosol (pneumonic), flea vectors (bubonic)	∼100% (pneumonic); 30%–60% (bubonic)	High: Person-to-person spread via droplets; flea/vector engineering feasible; state programs (e.g., WWII Japan)
	*Francisella tularensis* (Tularemia)	Aerosol, arthropod bites, contaminated water/food	∼35% (inhalation); 5%–15% (ulceroglandular)	High: Extremely low infectious dose (≤10 organisms); environmental stability; potential for aerosolized dissemination
	*Clostridium botulinum* (Botulism)	Foodborne, aerosol (toxin), wounds	∼60%–100% (without respiratory support)	High: Most potent toxin known (lethal dose ∼1 µg); scalable production; no natural immunity in humans
B (Moderate risk: moderately easy dissemination, moderate morbidity)	*Brucella spp*. (Brucellosis)	Contaminated food/dairy (fecal-oral), inhalation, contact with infected animals	<5% acute; chronic disability common (e.g., undulant fever)	Moderate: Zoonotic persistence in livestock; food supply contamination; lower mortality but economic/agricultural disruption
C (Emerging threats: potential for high impact via engineering)	None specifically discussed in this review	N/A	N/A	N/A (e.g., future synthetic biology-modified strains could elevate Category B agents to A-level threats)

In recent years, national investments in understanding infectious bacteria and their potential applications in BW have grown significantly, driven by both public health concerns and strategic military interests. The United States leads in annual funding, with the National Institutes of Health and the Defense Advanced Research Projects Agency allocating billions annually estimates suggest over $2 billion per year is directed toward biodefense and pathogen research (Gronvall, 2017). Other major players include China, Russia, and the United Kingdom, which have also expanded their biosecurity and dual-use research programs (Leitenberg and Zilinskas, 2012). Advances in synthetic biology and CRISPR-based genome editing have accelerated the ability to engineer pathogens, raising concerns about the weaponization of infectious agents (Esvelt and Gemmell, 2017). In modern warfare, the nation that dominates the synthesis, production, and strategic deployment of biological agents could gain a decisive tactical advantage, necessitating robust international oversight to prevent misuse (Tucker, 2012).

In the 21st century, the risk of bioterrorism persists, particularly with advancements in synthetic biology that could enable the engineering of antibiotic-resistant or vaccine-evading bacterial strains (DiEuliis and Giordano, 2017). The increasing accessibility of genomic editing tools, such as CRISPR-Cas systems, has sparked debate regarding the potential democratization of bioweapon capabilities, as these technologies could, in theory, be repurposed to enhance pathogen virulence or stability (Gootenberg et al., 2017). However, it is important to note that assessments of this risk vary, with some analyses suggesting a limited near-term uplift in biological weapons capabilities from these technologies (Paris, 2022). Simultaneously, globalization and urbanization facilitate the rapid spread of intentionally released pathogens, complicating containment efforts and amplifying potential casualties (Boyd et al., 2020). These factors necessitate a paradigm shift in biodefense strategies, integrating surveillance, diagnostics, and rapid response mechanisms.

Efforts to counter bacterial BW hinge on interdisciplinary innovations. Molecular modeling of *B. anthracis* enzymes, such as dihydrofolate reductase, has identified novel inhibitors to circumvent antibiotic resistance (Giacoppo et al., 2015), Additionally, clay-based decontaminants functionalized with quaternary ammonium salts neutralize *B. anthracis* spores on infrastructure surfaces, offering rapid remediation (Plachá et al., 2014). Moreover, computational prediction of conserved antigens in bacterial bioweapons, such as outer membrane proteins of *Y. pestis* and *Burkholderia pseudomallei*, aims to expedite universal vaccine development (Jaiswal et al., 2014).

Despite these advancements, significant challenges remain. The genetic plasticity of bacterial pathogens complicates the durability of countermeasures, necessitating continuous surveillance of emerging strains (Fouet and Mock, 2006). Furthermore, international disparities in biosecurity infrastructure and the dual-use nature of research necessitate robust ethical frameworks and global cooperation (Bakanidze et al., 2010). The 2001 anthrax attacks revealed gaps in diagnostic readiness and communication, underscoring the need for harmonized protocols across public health and law enforcement agencies (Jernigan et al., 2001).

This review examines the mechanisms by which infectious bacteria are weaponized, evaluates historical and contemporary case studies, and synthesizes current strategies for detection, prevention, and response. By contextualizing these elements within the evolving landscape of bioterrorism and biotechnology, this analysis aims to inform policy and research priorities to fortify global resilience against bacterial BW threats.

## 2 Types of bacteria used in biological warfare

### 2.1 High-threat agents

Category A BW agents are characterized by high mortality rates, ease of dissemination, and potential to cause public panic. Among these, *B. anthracis*, *Y. pestis*, and *F. tularensis* are prioritized due to their robustness, infectiousness, and historical use in biowarfare. These pathogens exploit aerosol transmission routes, evade host defenses, and resist environmental degradation, making them formidable threats (Inglesby et al., 2002; Dennis et al., 2009).

#### 2.1.1 *Bacillus anthracis* (anthrax)

*B. anthracis*, the causative agent of anthrax, poses a significant bioweapon threat due to its spore-forming capability, which ensures environmental persistence and resistance to heat, radiation, and disinfectants (Setlow, 2016; Dittmann et al., 2015). Spores exhibit crystalline nucleoid structures that protect DNA from stressors, while extracellular polysaccharides enhance adhesion to host tissues (Li et al., 2016). Inhalational anthrax, the most lethal form, rapidly disseminates from lungs to lymph nodes via CD11c+ cells, leading to systemic infection and high mortality within days (Shetron-Rama et al., 2010; Gutting et al., 2013).

Advanced detection methods, such as europium-based luminescent sensors and loop-mediated isothermal amplification (LAMP), enable rapid identification of anthrax biomarkers like dipicolinic acid (Abbas and Patra, 2022; Upadhyay et al., 2021). Electrochemical biosensors and γ phage amplification further improve diagnostic specificity (Raveendran et al., 2016; Cox et al., 2015). Despite these advances, challenges remain in decontaminating spore-laden environments. Chemical disinfectants (e.g., alcohols, quaternary ammonium compounds) are largely ineffective against resilient *B. anthracis* spores, necessitating U.S. environmental protection agency (EPA) registered sporicidal agents for reliable inactivation (Wood et al., 2021; Scorpio et al., 2007). Few antimicrobial pesticides are approved for *B. anthracis* spore decontamination, including chlorine dioxide-based solutions and hydrogen peroxide vapor; notably, one approved fumigant (e.g., methyl bromide) requires specialized application by certified vendors, complicating rapid response (Plachá et al., 2014; Wood et al., 2021; Scorpio et al., 2007). Dry thermal treatment at 250 °C effectively inactivates surface-bound spores but faces scalability limitations in large-scale scenarios (Wood et al., 2021).

Vaccine development focuses on enhancing protective antigen (PA) efficacy through TLR4/5 agonists and RNA aptamers targeting ribosomal proteins (Narayan et al., 2023; Pant, 2024). Adjuvants like CpG 7909 amplify immune responses to the BioThrax® vaccine, yet clarithromycin resistance mutations in *B. anthracis* underscore the need for novel antimicrobial strategies (Rynkiewicz et al., 2011; Maxson et al., 2024).

#### 2.1.2 *Yersinia pestis* (plague)

*Y. pestis*, responsible for pneumonic plague, has evolved from an enteric pathogen to a flea-borne and aerosol-transmitted biothreat (Sun et al., 2014). Pneumonic plague facilitates person-to-person transmission via respiratory droplets, with mortality approaching 100% if untreated (Dennis et al., 2009). The phospholipase D (Ymt) and Pla protease genes enhance survival in flea vectors and human hosts, respectively, by disrupting biofilm formation and manipulating host coagulation pathways (Jarrett et al., 2024; Wu et al., 2024).

Host-pathogen interactions reveal that *Y. pestis* evades innate immunity by inhibiting neutrophil recruitment and subverting platelet function to escape thrombi entrapment (Palace et al., 2020; KuoLee et al., 2011). Transcriptomic studies highlight the detrimental role of RIG-I-like receptor signaling during infection, which exacerbates inflammatory responses (Du et al., 2014). Bubonic plague models demonstrate rapid bacterial migration to lymph nodes via S1P-dependent mechanisms, mirroring human disease progression (John et al., 2014).

Diagnostic challenges persist due to non-specific early symptoms, but portable PCR platforms enable field detection (Molsa et al., 2015). Live attenuated vaccines, such as *Y. pseudotuberculosis*-based candidates, elicit robust cellular immunity but require optimization for safety (Derbise et al., 2025). Prairie dog studies emphasize the importance of vector control and vaccination during outbreaks, though ecological complexities hinder eradication (Eads et al., 2024).

#### 2.1.3 Francisella tularensis (tularemia)

*F. tularensis*, a facultative intracellular pathogen, is among the most infectious bacteria, requiring as few as 10 aerosolized organisms to cause disease (McLendon et al., 2006). Its lipopolysaccharide (LPS) structure, modified by late acyltransferases like LpxL, minimizes immune recognition, facilitating macrophage parasitism (McLendon et al., 2007; Benziger et al., 2023). Inhalational exposure triggers alveolar epithelial cell apoptosis suppression and macropinocytosis-mediated bacterial uptake, promoting systemic spread (Bradburne et al., 2013; Hutt et al., 2017).

Host immune evasion is mediated by *Francisella* pathogenicity island proteins, which delays apoptosis and inhibits pro-inflammatory cytokine production (Mahawar et al., 2012). Gender-specific vaccine responses in mice reveal heightened protection in females, linked to estrogen-enhanced Th1 immunity (Sunagar et al., 2016). The live vaccine strain (LVS) remains the gold standard but poses reactogenicity risks, driving efforts to develop subunit vaccines targeting outer membrane proteins (Mulligan et al., 2017).

Nanoprobe-based assays and TaqMan PCR improve detection sensitivity in clinical and environmental samples, critical for outbreak containment (Kim et al., 2015; Mitchell et al., 2010). However, environmental persistence in water and soil complicates decontamination, necessitating integrated biosurveillance strategies (Shang and Dando, 2023).

### 2.2 Secondary threats

#### 2.2.1 *Brucella* spp. (brucellosis)

Brucellosis, caused by *Brucella* spp., induces chronic granulomatous infections, leading to long-term disability and economic losses in livestock-dependent communities (Seleem et al., 2010). The pathogen’s ability to survive within macrophages via VirB-mediated type IV secretion system (T4SS) ensures systemic persistence (Celli, 2019). Recent meta-analyses highlight rising seroprevalence in Iran and the Middle East, linked to unpasteurized dairy consumption and occupational exposure (Dadar and Alamian, 2024; Dadar et al., 2025).

Diagnostic hurdles include cross-reactivity with other Gram-negative pathogens, addressed by LAMP assays targeting *Brucella*-specific IS711 elements (Chen et al., 2025). Vaccine development focuses on divalent DNA constructs encoding L7/L12-Omp31 fusion proteins, which enhance Th1 responses in ruminants (Golshani et al., 2015). However, RB51 vaccine shedding in buffalo milk underscores zoonotic transmission risks, necessitating stringent biosafety measures (Dadar et al., 2024).

#### 2.2.2 *Clostridium botulinum* (botulism)

*C. botulinum* produces botulinum neurotoxin (BoNT), the most potent biological toxin known, with lethal doses as low as 1 µg (Balali-Mood et al., 2013). BoNTs block acetylcholine release at neuromuscular junctions, causing flaccid paralysis. Structural studies reveal pH-dependent assembly of toxin complexes, enabling endosome escape and cytosolic SNARE protein cleavage (Ma et al., 2014; Roja et al., 2023).

Detection relies on mass spectrometry and CRISPR-based assays, though environmental inhibitors complicate PCR accuracy (Plößl et al., 2022; Sheykholeslami et al., 2024). UV irradiation effectively inactivates spores in water, but foodborne outbreaks persist due to thermal resistance (Assal et al., 2023). Equine antitoxins remain the primary treatment, but camelid-derived single-domain antibodies show promise for neutralizing BoNT/A (Hu et al., 2025). Recombinant bacterin vaccines reduce cattle mortality, yet lack cross-serotype protection (Moreira et al., 2020). *C. botulinum* poses risks due to toxin resistance. Table 2 summarizes key characteristics of these threats.

**TABLE 2:** High-threat bacterial bioweapons characteristics and virulence mechanisms.

Pathogen	Disease	Infectious dose	Primary transmission	Key virulence mechanisms	Mortality (untreated)
*B*. *anthracis*	Anthrax	8,000–50,000 spores	Aerosol, cutaneous	Spore resilience, poly-γ-D-glutamate capsule (immune evasion), lethal/edema toxins (MAPK disruption)	∼100% (inhalation)
*Y*. *pestis*	Plague	10–100 bacteria	Aerosol, flea vectors	F1 capsule anti-phagocytic, T3SS effectors (Yops), Pla protease (tissue invasion)	∼100% (pneumonic)
*F*. *tularensis*	Tularemia	≤10 bacteria	Aerosol, arthropods	LPS modifications (immune evasion), FPI-encoded T6SS (intracellular survival), macropinocytosis	∼35% (inhalation)
*Brucella* spp.	Brucellosis	1,000–10,000 bacteria	Contaminated food/dairy	VirB T4SS (intramacrophage survival), chronic granulomatous infection	<5% (chronic disability)
*C. botulinum*	Botulism	1 µg (toxin)	Food, aerosol	BoNT (paralysis)	∼60–100% (without respiratory support)

## 3 Transmission methods

### 3.1 Natural routes of dissemination

#### 3.1.1 Aerosolization (e.g., anthrax spores, pneumonic plague)

Natural aerosol transmission is a significant route for *B. anthracis* spores, which can remain viable in the atmosphere for extended periods and penetrate deep into the respiratory tract when inhaled. Studies simulating the release of spores from contaminated letters demonstrated that even minor mechanical disturbances, such as opening an envelope, generate respirable aerosols (1–5 μm in diameter) capable of dispersing thousands of spores into indoor environments (Kournikakis et al., 2009). Computational fluid dynamics models further quantified spore deposition in human and animal airways, revealing that particle size and airflow dynamics critically determine pulmonary retention and infection likelihood (Kabilan et al., 2016). This method of dissemination exploits the environmental persistence of spores, which can remain infectious for decades under dry conditions, enabling covert attacks with delayed onset of symptoms.

The epidemiological threat of aerosolized anthrax is amplified by its high infectivity and rapid disease progression. Inhalational anthrax, characterized by spore germination in lung-associated lymph nodes, leads to systemic toxin release and mortality within days if untreated. Primate studies using pulmonary spore challenges demonstrated that as few as 1,000–3,000 spores can establish lethal infections, with lethal toxin (LT) driving vascular collapse and multi-organ failure (Hutt et al., 2014). Historical analyses of bioterrorism events, such as the 2001 U.S. anthrax letters, highlighted the challenges of detecting low-dose aerosol exposures, as spores can evade conventional air sampling systems while contaminating broad spatial areas (Ho and Duncan, 2005). These findings underscore the dual risk of aerosolized anthrax: its capacity to cause mass casualties and overwhelm healthcare infrastructure during covert releases.

Mitigating aerosol-based threats requires interdisciplinary strategies spanning early detection, decontamination, and prophylactic countermeasures. Advances in sensor technologies, such as europium-doped fluorescence probes and competitive coordination assays, now enable rapid identification of dipicolinic acid a biomarker of bacterial spores in environmental samples (Su et al., 2021; Li et al., 2024). Concurrently, fabric-based filtration systems demonstrated >90% capture efficiency for aerosolized spores, providing passive protection in high-risk settings (Sajo et al., 2015). Vaccines like AV7909, designed for thermostability and rapid antibody induction, have shown efficacy in pre- and post-exposure prophylaxis (PEP) models, neutralizing toxins before systemic dissemination (Savransky et al., 2017). However, gaps persist in real-time aerosol monitoring and large-scale decontamination protocols, necessitating further innovation to address the evolving biothreat landscape.

#### 3.1.2 Contaminated food/water (e.g., *Brucella* in dairy)

The transmission of pathogenic bacteria through contaminated food and water remains a critical pathway for both natural outbreaks and potential exploitation in BW. *Brucella* spp., particularly *B. abortus* and *B. melitensis*, exemplify this threat due to their persistence in dairy products and ability to cause severe zoonotic infections. These bacteria are primarily disseminated through the consumption of unpasteurized milk, cheese, and other dairy products derived from infected livestock. For example, Marouf et al. (2021) demonstrated that *Brucella* spp. were prevalent in 14% of raw milk and artisanal cheese samples in Iran, highlighting the role of unregulated dairy production in sustaining transmission. Similarly, Gharekhani et al. (2021) identified herd-level contamination of milk in Iranian dairy farms, where poor sanitation and direct animal-to-milk contact facilitated bacterial dissemination. Such findings underscore the vulnerability of food supply chains to contamination, particularly in regions with limited adherence to pasteurization protocols.

The resilience of *Brucella* in environmental matrices further exacerbates its transmission risk. Sharma et al. (2024) provided evidence of *Brucella* excretion in cattle feces in Punjab, India, suggesting that contaminated water sources and soil could indirectly introduce the pathogen into dairy products through fecal-oral routes or agricultural runoff. This environmental persistence, combined with the global trade of dairy products, creates opportunities for widespread dissemination. Jansen et al. (2019) documented the illegal sale of *Brucella*-positive raw milk cheese in European markets, emphasizing how lax regulatory oversight and cross-border trade could amplify outbreaks. In a biowarfare context, deliberate contamination of dairy supplies in non-endemic regions could mimic natural transmission patterns, complicating attribution and enabling covert attacks on food security and public health.

Advanced detection methods have revealed the limitations of traditional surveillance in mitigating these risks. While real-time PCR assays (Milton et al., 2023) and surface plasmon resonance aptasensors (Dursun et al., 2022) enable rapid identification of *Brucella* in milk, many high-risk regions still rely on outdated cultural assays, delaying outbreak responses. Matope et al. (2010) identified herd management practices in Zimbabwean smallholder farms such as shared grazing and untreated water use as key contributors to *Brucella* seropositivity, illustrating how socioeconomic factors perpetuate transmission. To counter biothreats, strengthening food safety infrastructure, enforcing pasteurization mandates, and deploying portable detection technologies are critical to disrupt both natural and intentional dissemination routes.

#### 3.1.3 Vector-borne (e.g., fleas for plague)

Vector-borne transmission plays a critical role in the dissemination of bacterial pathogens, particularly in zoonotic diseases where arthropods serve as intermediaries. A prominent example is the transmission of *Y. pestis*, the causative agent of plague, by fleas such as *Xenopsylla cheopis* and *Ctenocephalides felis*. Fleas acquire *Y. pestis* during blood meals from infected mammalian hosts, enabling the bacteria to colonize the flea’s proventriculus and midgut (Kirillina et al., 2004; Forman et al., 2006; Bobrov et al., 2008). This colonization triggers biofilm formation, a process mediated by the *hms* gene cluster, which obstructs the flea’s digestive tract and forces regurgitation of bacteria-laden blood into new hosts during subsequent feeding. This mechanism ensures efficient transmission even at low bacterial loads, highlighting the evolutionary adaptation of *Y. pestis* to exploit flea vectors (Sun et al., 2014; Pisarenko et al., 2021).

Ecological and climatic factors significantly influence flea population dynamics and pathogen transmission efficiency. Studies in California and Kazakhstan demonstrated that temperature and humidity modulate flea abundance and activity, with warmer, arid conditions favoring flea survival and *Y. pestis* persistence in rodent reservoirs. Spatial modeling further revealed that plague outbreaks correlate with high rodent density and fragmented habitats, which enhance flea-host interactions (Buller et al., 2024; Laperrière et al., 2016). Additionally, precipitation patterns indirectly affect transmission; increased rainfall boosts vegetation growth, supporting larger rodent populations, while droughts force rodents into clustered refuges, amplifying flea-borne pathogen spread (Biggins et al., 2021; Russell et al., 2021).

Mitigating flea-mediated transmission requires integrated strategies. Field trials in North America showed that oral baits laced with fipronil reduced flea burdens on prairie dogs by 95%, disrupting *Y. pestis* transmission cycles (Poché et al., 2020). Similarly, recombinant subunit vaccines targeting *Y. pestis* F1 and V antigens induced robust immune responses in rodents, reducing mortality during experimental challenges (Goldberg et al., 2021). However, challenges persist, such as flea resistance to insecticides and the emergence of *Y. pestis* strains with altered virulence profiles. Surveillance of flea-associated pathogens, including *Bartonella* spp. and *Rickettsia felis*, is equally critical, as co-infections in fleas complicate diagnostics and amplify zoonotic risks (Zurita et al., 2024; Sidhoum et al., 2024).

### 3.2 Person-to-person spread

#### 3.2.1 Critical for *Yersinia pestis* (pneumonic plague) and rare in *Bacillus anthracis*

*Y. pestis*, the causative agent of pneumonic plague, disseminates primarily via respiratory droplets and aerosolized particles during close contact with infected individuals. Studies using murine models and *in vivo* imaging systems have demonstrated that *Y. pestis* colonizes the lower respiratory tract, leveraging virulence factors such as the *ail* gene product to adhere to alveolar epithelial cells and evade phagocytosis (Eichelberger et al., 2020; Zhang et al., 2020). The pathogen’s ability to suppress innate and adaptive immune responses including inhibition of neutrophil recruitment and cytokine signaling facilitates systemic spread from the lungs to lymph nodes and other organs (Yang et al., 2017; Venugopal and Pechous, 2024). This rapid dissemination underscores the pathogen’s adaptation to respiratory transmission, making pneumonic plague the most contagious and lethal form of the disease.

Person-to-person transmission is a hallmark of pneumonic plague, driven by the release of bacteria-laden respiratory droplets during coughing or sneezing. Research highlights the critical role of bacterial load and proximity in transmission efficiency, with studies in rat models showing that untreated pneumonic plague leads to 100% mortality within 48–72 h post-exposure (Kilgore et al., 2024). The absence of robust early immune detection due to *Y. pestis*’s suppression of TLR4 signaling and interferon responses enables unchecked bacterial replication in the lungs, amplifying transmission risk (Yang et al., 2017; Venugopal and Pechous, 2024). This contrasts sharply with bubonic plague, where transmission typically requires flea vectors, emphasizing the unique public health threat posed by respiratory spread.

In contrast to *Y. pestis*, *B. anthracis,* the agent of anthrax, is not transmitted between humans, even in pulmonary cases (Ashique et al., 2024). Anthrax infections primarily arise from environmental exposure to spores through contaminated soil, animal products, or intentional release (Matero et al., 2011; Albrecht et al., 2012). Unlike *Y. pestis*, *B. anthracis* does not colonize the respiratory mucosa or produce adhesins that facilitate direct human-to-human transfer. Clinical cases of inhalational anthrax exhibit low bacterial shedding, and no documented instances of secondary transmission exist in modern literature (Raoult et al., 2013). The pathogen’s reliance on spore dormancy and environmental persistence, rather than active host-to-host dissemination, explains this epidemiological distinction.

The transmission methods of *Y. pestis* and *B. anthracis* reflect their divergent evolutionary strategies. *Y. pestis* rapid adaptation to respiratory niches and immune evasion mechanisms make person-to-person spread central to pneumonic plague outbreaks. In contrast, *B. anthracis* dependence on environmental reservoirs and lack of mucosal tropism limit its capacity for human transmission. These differences underscore the necessity of tailored public health interventions: rapid containment for pneumonic plague and environmental decontamination for anthrax.

## 4 Molecular and immunological basis of bacterial pathogenicity

High-consequence bacterial agents relevant to BW share a convergent pathogenic logic: efficient host entry (often via inhalation), rapid amplification in permissive niches, early suppression of innate sensing, and progression to severe disease through immune subversion and/or toxins. Across *Bacillus anthracis*, *Yersinia pestis*, and *Francisella tularensis*, virulence is typically not driven by a single determinant but by coordinated mechanisms that delay recognition, blunt clearance, and accelerate tissue injury features that shorten the window for clinical detection and effective intervention following exposure (McLendon et al., 2006; Jelacic et al., 2014; Agrawal and Pulendran, 2004; Du et al., 2002).

### 4.1 Immune evasion as a force multiplier for BW relevance

A defining feature of BW-relevant bacteria is the ability to delay or disable first line defenses long enough to establish systemic infection. This delay increases the likelihood that a low dose exposure progresses to severe disease before pathogen specific immunity, diagnosis, or antimicrobial therapy can be deployed.

#### 4.1.1 Anti-phagocytic capsules and low-immunogenic stealth surfaces

*B. anthracis* produces a poly-γ-D-glutamic acid (PGA) capsule that is relatively poorly immunogenic and interferes with opsonophagocytic clearance, in part by limiting complement deposition and reducing effective uptake by professional phagocytes (Jelacic et al., 2014; Sharma et al., 2020; Jelacic et al., 2021; Jang et al., 2011). Beyond simple “shielding,” capsule exposure can suppress dendritic-cell maturation and antigen presentation, weakening downstream T-cell priming and slowing the development of protective adaptive responses (Jelacic et al., 2014; Jelacic et al., 2021). Capsule production is tightly regulated by mammalian host cues (e.g., CO_2_/bicarbonate), reinforcing that these virulence traits are optimized for infection rather than environmental growth (Drysdale et al., 2005; Sittner et al., 2021). Importantly, enzymatic disruption of capsule anchoring and integrity (e.g., CapD-targeted approaches) can restore vulnerability to phagocytosis and killing, underscoring capsule-associated pathways as countermeasure targets (Scorpio et al., 2007; Candela and Fouet, 2005; Richter et al., 2009; Matharoo et al., 2022; Chen et al., 2011).

Similarly, *Y. pestis* expresses the F1 capsule at mammalian temperatures, limiting phagocytosis and contributing to early persistence during infection (Du et al., 2002; Peters et al., 2022). In practical terms, capsule-mediated suppression of early clearance increases the risk that initial nonspecific symptoms progress rapidly to fulminant disease before clinical suspicion is raised particularly relevant for pneumonic plague scenarios (Du et al., 2002).

### 4.2 Intracellular survival and immune “silencing”

Some high-threat bacteria exploit intracellular lifestyles that reduce exposure to antibodies and complement while directly suppressing inflammatory signaling.

#### 4.2.1 Francisella tularensis as a stealth intracellular pathogen

*F. tularensis* exemplifies an intracellular strategy that combines rapid phagosomal escape with cytosolic replication, thereby avoiding lysosomal killing and dampening early inflammatory responses (Sjöstedt, 2006; Celli and Zahrt, 2013; Chong et al., 2008; Santic et al., 2005; Wehrly et al., 2009). Proteins encoded in the *Francisella* pathogenicity island are central to intracellular survival, enabling phagosomal escape and replication in a niche where humoral immunity has limited direct access (Celli and Zahrt, 2013; Barker et al., 2009; Robertson et al., 2013). Additional traits including O-antigen associated evasion of autophagy and broad metabolic adaptability support persistence in nutrient limited intracellular environments and prolong the interval before effective immune containment is achieved (Ziveri et al., 2017; Radlinski et al., 2018; Case et al., 2014; Steele et al., 2013). Collectively, these mechanisms help explain why early recognition and timely antimicrobial therapy are pivotal in tularemia preparedness (McLendon et al., 2006; Sjöstedt, 2006; Celli and Zahrt, 2013; Chong et al., 2008; Santic et al., 2005; Wehrly et al., 2009).

### 4.3 Effector-mediated immune subversion and dysregulated inflammation

Beyond several BW-relevant agents actively reprogram host immunity using specialized secretion systems or immunomodulatory factors. These mechanisms can disable phagocyte function, suppress cytokine signaling, and reshape cell-death pathways in ways that favor bacterial survival.

#### 4.3.1 Type III secretion and targeted disabling of innate defenses in plague

A central immunological advantage of *Y. pestis* is its type III secretion system (T3SS), which injects Yop effectors into host cells to disrupt actin dynamics and signaling pathways required for phagocytosis and inflammatory activation (Yang et al., 2011; Bohn et al., 2019). The organism also leverages immunomodulatory factors such as LcrV to bias responses toward IL-10 associated suppression, reducing microbicidal functions in macrophages (Sing et al., 2002; Weeks et al., 2002; Bergsbaken and Cookson, 2009). In parallel, YopM can inhibit inflammasome-driven IL-1β maturation and interfere with pyroptotic clearance pathways, limiting early containment and neutrophil recruitment (LaRock and Cookson, 2012; Chung et al., 2016). Mechanistically, these features help explain why pneumonic plague can progress rapidly: *Y. pestis* neutralizes innate mechanisms that would otherwise control early lung infection (Du et al., 2002; Yang et al., 2011; LaRock and Cookson, 2012).

#### 4.3.2 Immunopathology: when host response becomes part of the injury

Severe disease is not always a simple function of bacterial load. In pneumonic plague, lung injury can reflect both overwhelming replication and a maladaptive inflammatory response that culminates in necrotizing pneumonia and abrupt clinical deterioration (Olson et al., 2021; Zimbler et al., 2016; Agar et al., 2008; Wang et al., 2024; Peters et al., 2013; Parent et al., 2006). This dual contribution matters for preparedness because patients may transition quickly from nonspecific symptoms to respiratory failure, stressing triage, isolation capacity, and critical-care resources during clustered events (Olson et al., 2021; Wang et al., 2024). Thus, mechanistic understanding supports the epidemiological emphasis on rapid detection and immediate containment for transmissible respiratory plague scenarios (see Section 5).

### 4.4 Toxin-mediated pathogenesis and systemic collapse

Some high-threat bacterial syndromes are dominated by toxins that cause host damage disproportionate to bacterial burden and can remain clinically relevant even after antimicrobials are started.

#### 4.4.1 Anthrax toxins: immune dysfunction and vascular injury

Anthrax lethal toxin (LT) disrupts host signaling by proteolytically targeting MAPK pathway components, undermining cytokine responses, immune-cell function, and barrier integrity (Agrawal and Pulendran, 2004; Turk, 2007). Experimental work also indicates that LT impairs dendritic-cell function and adaptive immunity, weakening protective antigen presentation during a critical early window (Agrawal et al., 2003). Systemically, toxin effects contribute to vascular leakage and shock physiology, linking molecular mechanisms directly to the rapid lethality feared in inhalational anthrax (Ghosh et al., 2012). From a countermeasure perspective, studies suggesting benefit from protecting or restoring key signaling pathways (e.g., ERK pathway activity) reinforce toxin-linked signaling interference as an actionable therapeutic axis rather than a purely descriptive mechanism (Liu et al., 2025).

Anthrax-associated organ injury can extend beyond the respiratory tract, including hepatic involvement during systemic disease, consistent with the concept that toxins and immune dysregulation converge to produce multi-organ dysfunction rather than localized infection alone (Liu et al., 2013; Altunkaynak and Ozbek, 2015; Grinberg et al., 2001; Colliou et al., 2015).

#### 4.4.2 Botulinum neurotoxin: extreme potency with primarily physiologic lethality

BoNT illustrates how a single bacterial product can dominate clinical outcome. BoNT blocks acetylcholine release by cleaving SNARE proteins, producing flaccid paralysis and respiratory failure (Lonati et al., 2020; Rossetto et al., 2021). The prolonged activity of some serotypes increases demands for extended ventilatory support and critical care resources an important consequence management concern even when antimicrobials are irrelevant to toxin already internalized in neurons (Rummel, 2015; Pellett et al., 2018).

### 4.5 Virulence platforms and adaptability: implications for evolving threat scenarios

Across BW prioritized agents, virulence is often organized into modular platforms capsules, secretion systems, intracellular survival programs, and toxins. These platforms can be altered by natural selection and, in dual-use contexts, raise concerns about the durability of standard countermeasures.

#### 4.5.1 Adhesion and colonization modules

In *Y. pestis*, adhesins such as the pH6 antigen support tissue attachment and can interfere with opsonophagocytic processes, reinforcing early establishment in host tissues (Makoveichuk et al., 2003; Zav’yalov et al., 1996; Pakharukova et al., 2016). Because early colonization strongly shapes whether exposure becomes progressive infection, these front-end interactions are relevant targets for vaccines and immunotherapies.

#### 4.5.2 Genetic plasticity and resistance acquisition

Horizontal gene transfer enables acquisition of antibiotic resistance determinants via mobile elements and plasmids, eroding standard prophylaxis and treatment assumptions if resistant variants emerge (Arnold et al., 2022; Rodríguez-Beltrán et al., 2021; Lerminiaux and Cameron, 2019; Pfeifer et al., 2022). Biofilm-like contexts and stress exposures can further promote transfer and stabilization of resistance traits, emphasizing that countermeasure erosion is an ongoing biological reality rather than a theoretical edge case (de Sousa et al., 2023; Wang et al., 2023; Lee et al., 2022; Domenech et al., 2020; Lopatkin et al., 2017; Haudiquet et al., 2022). Furthermore, recent advances in biotechnology and synthetic biology have enabled the deliberate engineering of pathogenic microorganisms, altering their pathogenicity, host tropism, and environmental stability. These engineered modifications pose severe difficulties in responding to biothreats, as they can abruptly render standardized diagnostic protocols and stockpiled medical countermeasures ineffective. These mechanisms reinforce the need for surveillance strategies that can detect resistance and virulence shifts early (see Section 6.1; Table 5).

### 4.6 BW-relevant takeaways

The core biodefense insight from these mechanisms is that severe disease is driven by immune timing. High-threat bacteria (i) delay recognition and clearance through capsules and stealth surfaces (Jelacic et al., 2014; Du et al., 2002; Sharma et al., 2020; Jelacic et al., 2021; Jang et al., 2011; Peters et al., 2022), (ii) establish protected intracellular niches and suppress inflammatory signaling (Sjöstedt, 2006; Celli and Zahrt, 2013; Chong et al., 2008; Santic et al., 2005; Wehrly et al., 2009), (iii) actively disable innate pathways via injected effectors and immunomodulatory skewing (Yang et al., 2011; Bohn et al., 2019; Sing et al., 2002; Weeks et al., 2002; Bergsbaken and Cookson, 2009; LaRock and Cookson, 2012; Chung et al., 2016; Vadyvaloo et al., 2010), (iv) abruptly convert infection into systemic catastrophe through toxins and immunopathology (Agrawal and Pulendran, 2004; Olson et al., 2021; Zimbler et al., 2016; Agar et al., 2008; Wang et al., 2024; Peters et al., 2013; Parent et al., 2006; Turk, 2007; Agrawal et al., 2003; Ghosh et al., 2012; Liu et al., 2025). Framing pathogenesis around these themes clarifies why even limited exposures can have disproportionate clinical and public health consequences and why layered countermeasures must integrate rapid recognition, early effective therapy, and approaches that neutralize immune evasion and toxin action rather than relying on any single molecular target in isolation (Scorpio et al., 2007; LaRock and Cookson, 2012; Liu et al., 2025).

## 5 Spread rate and epidemiological impact

### 5.1 Factors influencing outbreaks

#### 5.1.1 Incubation period (e.g., rapid spread of pneumonic plague vs. slower brucellosis)

The incubation period the time between pathogen exposure and symptom onset critically shapes outbreak dynamics. Pathogens with short incubation periods, such as *Y. pestis* (causing pneumonic plague), facilitate rapid transmission due to quicker symptomatic expression and higher infectiousness. In contrast, pathogens like *Brucella* spp. (causing brucellosis) exhibit prolonged incubation periods, delaying detection and enabling silent spread through asymptomatic carriers.

Genomic studies further underscore the role of pathogen evolution in incubation dynamics. Xie et al. (2024) identified lineage-specific differences in *Streptococcus dysgalactiae* subsp. *equisimilis*, where certain clades exhibited faster dissemination due to virulence factors shortening the incubation period. These findings align with observations in *Y. pestis* outbreaks, where rapid progression to pneumonic phases fuels explosive spread. Collectively, these studies emphasize that incubation period heterogeneity demands tailored surveillance and containment strategies, prioritizing rapid diagnostics for short-incubation pathogens and serological monitoring for slower ones like *Brucella*.

#### 5.1.2 Population density and mobility

High population density and mobility amplify outbreak risks by increasing contact rates and pathogen dispersal. Urban environments, transportation hubs, and healthcare facilities serve as hotspots for transmission due to crowded conditions. For example, Gohli et al. (2019) found that subway systems host distinct microbial communities dominated by human-associated bacteria (e.g., *Staphylococcus*), with seasonal fluctuations in diversity linked to commuter density. Pathogen abundance rose during peak travel times, illustrating how mobility drives microbial exchange.

Human and animal mobility further enables long-distance pathogen spread. Kamath et al. (2016) reconstructed *Brucella* transmission between wildlife (elk) and livestock using genomics, revealing that migration patterns and shared grazing areas facilitated cross-species spillover. Similarly, Senghore et al. (2023) combined genomic surveillance with mobility data to map *Streptococcus pneumoniae* transmission networks in urban Gambia, showing that highly mobile individuals (e.g., market vendors) bridged geographically distinct clusters. These findings highlight the role of mobile populations as superspreaders in interconnected communities.

In healthcare settings, Frickmann et al. (2024) demonstrated that invasive ant species (*Lasius neglectus*) mechanically transported multidrug-resistant bacteria (e.g., *Enterococcus faecalis*) across hospital wards, exploiting high-density infrastructure. This underscores how even non-human vectors exacerbate risks in crowded environments. Mitigation requires spatial interventions such as decongesting transit systems, restricting livestock movement, and optimizing hospital layouts to disrupt transmission pathways amplified by density and mobility. These factors underpin outbreak potential. Table 3 compares epidemiological drivers.

**TABLE 3:** Epidemiological impact and outbreak drivers.

Factor	Anthrax	Pneumonic plague	Tularemia	Implications
Incubation period	1–6 days (inhalation)	1–3 days	3–5 days	Short incubation to Rapid outbreaks (plague) vs. delayed detection (anthrax)
Person-to-person	None	High	Rare	Plague requires rapid isolation; anthrax decontamination focus
Environmental persistence	Decades (spores)	Months (soil/flea vectors)	Weeks (water/soil)	Anthrax spores complicate long-term remediation
Global spread risk	Moderate (stability)	High (aerosol + human transmission)	Low (environmental decay)	Globalization amplifies plague/brucellosis risks via travel/trade (Boyd et al., 2020; Jansen et al., 2019)

### 5.2 Case studies

#### 5.2.1 2001 U.S. anthrax attacks: limited spread due to non-contagious nature

The 2001 U.S. anthrax attacks highlighted the unique challenges and advantages of responding to a non-contagious bioterrorism agent. *B. anthracis*, the causative bacterium, lacks person-to-person transmissibility, which confined infections to direct spore exposure and simplified containment efforts. Updated CDC guidelines (Bower et al., 2023) emphasize that rapid antibiotic prophylaxis (e.g., ciprofloxacin) and environmental decontamination prevented secondary cases, with no evidence of human transmission. Eisenkraft et al. (2018) validated this approach using cell phone mobility data to simulate outbreak scenarios, demonstrating how targeted contact tracing could isolate exposures within 48 h. These findings confirm that non-contagious agents limit epidemic potential but demand precise epidemiological tools for effective mitigation. From a security perspective, the 2001 incident also triggered a massive proliferation of copycat crimes and pranks, which caused widespread chaos and severely overwhelmed diagnostic testing facilities. Furthermore, because this incident is suspected to be an insider crime, it underscores the paramount importance of rigorous personnel background checks, continuous biosecurity training, and strict codes of conduct to prevent malevolent acts by individuals with authorized access.

Post-2001 research prioritized predictive modeling and surveillance to address gaps in bioterrorism preparedness. Rainisch et al. (2017) developed a decision-support model projecting 10,000–100,000 potential cases in a large-scale aerosolized release, underscoring the need for preemptive stockpiling of medical countermeasure (MCM). Vieira et al. (2017) further advocated for integrating genomic surveillance into national networks to detect engineered strains, a strategy now embedded in U.S. biodefense protocols. Collectively, these studies demonstrate that combining real-time data analytics with robust surveillance can reduce mortality even in high-risk scenarios. However, the remediation phase following incidents like the 2001 attacks involved extensive decontamination efforts spanning months and costing millions of dollars, highlighting the persistent challenges in environmental cleanup and recovery. For example, the decontamination of the U.S. Department of Justice mail facility required complex sporicidal protocols and prolonged infrastructure remediation, revealing gaps in scalable response frameworks for *Bacillus anthracis* spore persistence (Franco and Bouri, 2010; Canter, 2005).

#### 5.2.2 Hypothetical plague release: high mortality and healthcare collapse

A deliberate release of *Y. pestis*, the plague bacterium, represents a catastrophic bioterrorism threat due to its high fatality rate and rapid transmission. Tin et al. (2022) classifies pneumonic plague as a Tier 1 agent, with untreated mortality exceeding 90% within 18–24 h of symptom onset. Cieslak et al. (2018) estimates that a single aerosolized release in a major city could infect 150,000 individuals, overwhelming ICU capacity and antibiotic supplies within days. Historical parallels, such as the 1994 Surat outbreak, illustrate how delayed diagnosis and inadequate isolation protocols can accelerate healthcare system failure.

Neuroinvasive complications and triage dilemmas further exacerbate mortality risks. Ralston et al. (2019) identifies plague’s potential to induce septic shock and meningitis, requiring specialized neurocritical care unavailable in most hospitals during mass outbreaks. Zhao et al. (2023) stresses that crisis standards of care prioritizing antibiotics for patients with ≤48-h survival likelihood could reduce mortality by 15%–20% during resource triage. However, without preemptive stockpiling of doxycycline and ventilators, these strategies remain theoretical. Research unanimously concludes that plague bioterrorism would precipitate unparalleled mortality without multi-layered preparedness frameworks.

## 6 Defense strategies and future directions

### 6.1 Detection and surveillance

#### 6.1.1 Rapid diagnostics (PCR, CRISPR-based tools)

The rapid identification of bacterial biothreat agents has been revolutionized by advancements in PCR-based diagnostics, which remain the gold standard for sensitivity and specificity. Molsa et al. (2015) demonstrated the utility of portable real-time PCR systems for detecting *F. tularensis*, *B. anthracis*, and *Y. pestis* in under 90 min, achieving limits of detection (LOD) comparable to laboratory-grade instruments. This portability is critical for field deployment during biothreat outbreaks. Similarly, Wilson et al. (2005) developed a multiplex PCR-coupled liquid bead array to simultaneously detect *B. anthracis*, *Y. pestis*, *F. tularensis*, and *Brucella melitensis*, minimizing cross-reactivity and enabling high-throughput screening. Sampath et al. (2012) further expanded multiplex capabilities by integrating PCR with electrospray ionization mass spectrometry (PCR/ESI-MS), allowing simultaneous identification of 16 biothreat pathogens, including *Burkholderia* and *Brucella* species, in clinical and environmental matrices. These studies underscore PCR’s adaptability for both targeted and broad-spectrum pathogen detection in biodefense scenarios.

Recent innovations in PCR methodologies have focused on enhancing speed, quantification, and viability assessment. Du et al. (2022) developed a multiplex droplet digital PCR (ddPCR) assay for five high-risk pathogens (*B. anthracis*, *Y. pestis*, *F. tularensis*, *Brucella*, and *Burkholderia*), achieving an LOD of 10 copies/μL with absolute quantification, even in complex backgrounds. This approach outperformed traditional qPCR in accuracy and resistance to inhibitors. Meanwhile, Létant et al. (2011) addressed a critical gap in environmental monitoring by combining propidium monoazide (PMA) pretreatment with PCR to distinguish live *B. anthracis* spores from non-viable remnants, a necessity for assessing active threats. Complementary to this, rapid viability PCR (RV-PCR) employs a brief culture step followed by automated DNA extraction and pathogen-specific PCR, enabling high-throughput, culture-confirmed detection of viable *B. anthracis* spores with high specificity, significantly accelerating clearance assessments after decontamination (Kane et al., 2009; Kane et al., 2013; Brisson et al., 2025). Egbo et al. (2025) optimized LAMP for *B. anthracis* and *Y. pestis*, though noted variability in clinical sensitivity compared to PCR. These advancements highlight the evolving role of PCR-derived technologies in balancing rapidity with analytical rigor.

CRISPR-based diagnostics have emerged as transformative tools, offering equipment-free workflows and single-molecule sensitivity. Bentahir et al. (2018) integrated recombinase polymerase amplification (RPA) with CRISPR-Cas12a to detect *B. anthracis* in under 30 min, achieving an LOD of 10 CFU/mL while differentiating virulent strains from non-pathogenic *Bacillus* species. Saxena et al. (2019) adapted this system for *Burkholderia mallei*, pairing RPA-CRISPR with lateral flow assays for visual readouts in resource-limited settings. Turingan et al. (2013) leveraged CRISPR-Cas9 for strain-level resolution, targeting single-nucleotide polymorphisms in *B. anthracis* and *Y. pestis* to support both diagnostics and forensic tracing. Gunnell et al. (2012) further demonstrated the versatility of CRISPR by coupling it with multiplex PCR for *Francisella tularensis* detection in metagenomic samples, enhancing specificity in polymicrobial environments. These platforms exemplify CRISPR’s potential to bridge the gap between laboratory precision and field applicability.

Hybrid approaches combining PCR and CRISPR technologies are increasingly addressing limitations of standalone methods. Yang et al. (2009) paired universal PCR with high-resolution melting analysis to differentiate biothreat agents such as *B. anthracis* and *Y. pestis* from clinically similar species, reducing false positives in mixed samples. Jeng et al. (2013) expanded this concept using RT-PCR/ESI-MS to simultaneously identify bacterial and viral pathogens, demonstrating utility in respiratory infection surveillance. Rajan et al. (2022) highlighted the adaptability of CRISPR-Cas13-based SHERLOCK assays, originally designed for viral RNA, for bacterial DNA detection through modular guide RNA redesign. These integrative strategies enhance diagnostic breadth while maintaining rapid turnaround times, critical for both clinical and biodefense applications.

Despite these advancements, challenges persist in standardization, cost, and real-world validation. Egbo et al. (2025) noted that LAMP assays, while rapid, require rigorous optimization to match PCR’s consistency across diverse sample types. Similarly, CRISPR platforms face hurdles in clinical translation due to interference from host DNA and complex sample matrices, as observed by Rajan et al. (2022). Future efforts must prioritize harmonizing CRISPR workflows with existing biosurveillance networks and validating assays against environmental and clinical specimens. The integration of machine learning for primer design and automated sample preparation could further enhance both PCR and CRISPR systems. However, while targeted techniques like PCR and CRISPR are highly effective for known agents, they may fail to detect pathogens that have undergone unfamiliar mutations or deliberate genetic modifications. To deal with these threats, biosurveillance frameworks must increasingly integrate agnostic diagnostic approaches, such as next-generation sequencing and metagenomics, which can identify engineered sequences and novel virulence traits without relying on predefined targets. Collectively, these technologies represent complementary pillars in the rapid diagnostics landscape, each addressing unique operational demands in the containment of bacterial biothreats.

### 6.2 Medical countermeasures

#### 6.2.1 Vaccines (e.g., anthrax vaccine AVA)

Anthrax vaccines remain critical for PEP against *B. anthracis*, a high-priority bioterrorism agent. The licensed Anthrax Vaccine Adsorbed (AVA, BioThrax®), composed of PA from the anthrax toxin, has been the cornerstone of vaccination strategies. Clinical studies demonstrate that intramuscular (IM) administration of AVA induces robust IgG responses to PA, with seroconversion rates exceeding 95% after three doses (Pittman et al., 2002; Singer et al., 2008). Reduced-dose regimens (e.g., 3-dose IM schedules) retain immunogenicity while minimizing adverse events, such as injection-site reactions, and provide sustained protection for ≥3 years (Marano et al., 2008; Quinn et al., 2012). Updated guidelines from the Advisory Committee on Immunization Practices recommend AVA for high-risk populations, including military personnel and laboratory workers, with booster doses every 3 years (Bower, 2019). However, challenges persist in optimizing PEP efficacy, as delayed dosing reduces antibody titers, necessitating strict adherence to vaccination schedules (Pittman et al., 2013).

Second-generation vaccines, such as AV7909 (AVA + CpG 7909 adjuvant), aim to enhance immunogenicity and accelerate protection. In preclinical models, two AV7909 doses administered 2 weeks apart conferred 100% survival in guinea pigs and nonhuman primates (NHPs) exposed to aerosolized *B. anthracis*, with neutralizing antibody titers exceeding thresholds for protection (Shearer et al., 2021; Henning et al., 2023). Lyophilized AV7909 retains stability and potency under temperature fluctuations, addressing logistical challenges in biodefense stockpiling (Smiley et al., 2019). Phase I trials in humans confirm its safety, with transient local reactions comparable to AVA, and adjuvantation with CpG 7909 significantly boosts anti-PA IgG and toxin-neutralizing capacity (Hopkins et al., 2013). These advances position AV7909 as a promising candidate for rapid PEP deployment.

Immune correlates of protection highlight the critical role of anti-PA IgG subclasses, particularly IgG1 and IgG3, in toxin neutralization and opsonophagocytic activity. AVA-vaccinated individuals and anthrax survivors exhibit similar IgG subclass profiles, suggesting functional mimicry of natural immunity (Semenova et al., 2007). In rhesus macaques, anti-PA IgG titers ≥200 μg/mL correlate with 100% survival against lethal inhalational challenge, providing a benchmark for vaccine efficacy (Chen et al., 2014). Notably, co-administration of monoclonal antibodies (e.g., raxibacumab) with AVA does not impair immunogenicity, enabling synergistic post-exposure strategies (Skoura et al., 2020). Conversely, concurrent ciprofloxacin use with AVA slightly delays seroconversion but does not compromise long-term antibody production, supporting antibiotic-vaccine coadministration during PEP (Cassie et al., 2024).

Animal models, particularly guinea pigs and NHPs, underpin anthrax vaccine development. The guinea pig inhalational anthrax model demonstrates that AV7909 administered 24 h post-exposure achieves 80%–90% survival, outperforming AVA alone (Perry et al., 2020; Ionin et al., 2013). These findings align with NHP data, where early post-exposure vaccination primes humoral immunity to neutralize toxins before lethal bacteremia ensues. However, species-specific differences in immune responses such as guinea pigs’ reliance on antibody-mediated protection versus NHPs’ additional T-cell contributions highlight the need for complementary models (Chen et al., 2014).

Emerging approaches aim to broaden protection and simplify delivery. Nasal vaccines incorporating PA and LF antigens induce mucosal IgA and systemic IgG in preclinical studies, potentially blocking spore germination at respiratory entry points (Sloat and Cui, 2006). While these innovations remain experimental, they underscore the importance of next-generation platforms to address evolving biodefense threats. Collectively, AVA and AV7909 exemplify the integration of immunology, clinical practice, and biopreparedness in countering anthrax as a biological weapon.

#### 6.2.2 Antibiotics and antitoxins (e.g., ciprofloxacin for plague)

Fluoroquinolones, particularly ciprofloxacin, have demonstrated robust efficacy against *Y. pestis* in preclinical and clinical studies. In murine models of pneumonic plague, ciprofloxacin and doxycycline significantly increased survival rates when administered prophylactically or therapeutically (Russell et al., 1998; Russell et al., 1996). Ciprofloxacin’s ability to penetrate macrophages and target intracellular *Y. pestis* further underscores its utility, as the bacterium evades immune detection by surviving within host phagocytes (Wendte et al., 2011). These findings align with clinical evidence from human plague cases, where oral ciprofloxacin achieved 100% survival in culture-confirmed infections, even in advanced stages (Andrade et al., 2020). The African green monkey model, a gold standard for regulatory approval under the FDA’s Animal Rule, validated ciprofloxacin’s >90% efficacy when initiated within 42 h post-exposure, reinforcing its role as a frontline countermeasure (Campbell et al., 2020; Hewitt et al., 2020).

Combination therapies have emerged as a strategy to enhance therapeutic outcomes and mitigate resistance risks. In murine bubonic plague, the synergistic effect of ciprofloxacin and gentamicin reduced bacterial loads in lymph nodes and spleen more effectively than monotherapy, suggesting a potential role for dual regimens in severe infections (Lemaîtr et al., 2012). Similarly, *in vitro* pharmacodynamic models simulating human pharmacokinetics revealed that gentamicin and ciprofloxacin achieved near-complete bactericidal activity against *Y. pestis* within 24 h, outperforming β-lactams (Louie et al., 2011). Clinical trials in Madagascar further explored ciprofloxacin as a standalone or adjunct therapy, with non-inferiority to streptomycin-based regimens in treating bubonic plague, highlighting its versatility in resource-limited settings (Randremanana et al., 2020).

Novel antibiotics, including omadacycline and tebipenem, expand the arsenal against *Y. pestis*. Omadacycline, a tetracycline derivative, exhibited potent *in vitro* activity (MIC_90_ ≤ 0.25 μg/mL) and conferred 80%–100% survival in murine pneumonic plague models, even when treatment was delayed (Steenbergen et al., 2017). Tebipenem, an oral carbapenem, demonstrated efficacy equivalent to ciprofloxacin in African green monkey models, with survival rates exceeding 75% when administered 24 h post-exposure (Clayton et al., 2021). These agents address critical gaps, particularly for penicillin-allergic patients or scenarios requiring oral administration. Additionally, GT-1, a siderophore cephalosporin, showed promising *in vivo* activity against *Y. pestis*, suggesting a role for iron-uptake-targeted therapies in overcoming bacterial evasion mechanisms (Halasohoris et al., 2021).

Timely intervention remains pivotal, as delayed antibiotic administration drastically reduces efficacy. African green monkey studies revealed that ciprofloxacin’s survival benefit dropped from 90% to 25% when treatment began 42 versus 54 h post-infection (Campbell et al., 2020). Similarly, murine models showed that β-lactams like ceftazidime accelerated mortality due to toxin release from lysed bacteria, emphasizing the need for rapid, pathogen-specific diagnostics (Byrne et al., 1998). Innovations such as phage-based susceptibility assays now enable rapid (<6 h) determination of antibiotic efficacy, facilitating personalized treatment and outbreak containment (Moses et al., 2021).

While the development of novel medical countermeasures is paramount, it is important not to overlook the foundational relevance of traditional countermeasures. Standard antibiotics remain the primary treatment for infections caused by *Francisella tularensis*, *Clostridium botulinum* (e.g., for wound botulism management), and *Yersinia pestis*. Potential remedies for these weaponized organisms prominently include antibiotics derived from *Streptomyces* species, such as streptomycin and various tetracyclines. Since many of these severe bacterial pathogens originate in nature, further exploration of natural ecological sources and soil microbiota may yield crucial, additional countermeasures against both naturally emerging and engineered strains.

In conclusion, ciprofloxacin and doxycycline remain cornerstones of plague management, supported by robust preclinical and clinical data. Combination therapies and novel antibiotics broaden therapeutic options, while rapid diagnostics and adherence to early intervention protocols are critical to optimizing outcomes. These advances underscore the importance of integrating pharmacodynamic insights with clinical pragmatism to counter *Y. pestis* as a biothreat. Novel antibiotics highlight evolving countermeasures. Table 4 synthesizes advances and limitations.

**TABLE 4:** Advanced detection and medical countermeasures.

Technology/strategy	Application	Key advancements	Limitations
CRISPR-based diagnostics	Pathogen detection (e.g., *B. anthracis*, *Y. pestis*)	RPA-CRISPR (LOD: 10 CFU/mL), SHERLOCK assays (single-molecule sensitivity)	Host DNA interference, sample matrix complexity
Portable PCR/LAMP	Field identification of biothreats (*F. tularensis*, *B. anthracis*)	Multiplex ddPCR (LOD: 10 copies/μL), PMA-PCR (viability assessment)	Variable clinical sensitivity (LAMP), optimization needed
Decontamination strategies	Surface/environmental remediation	EPA-registered sporicides (ClO_2_, H_2_O_2_ vapor); clay-QAC composites (Plachá et al., 2014; Wood et al., 2021)	Few approved agents; fumigant dependency; material corrosion; scalability limits (Wood et al., 2021)
Vaccines	Pre/post-exposure prophylaxis	AV7909 (AVA + CpG 7909) (Shearer et al., 2021; Henning et al., 2023; Smiley et al., 2019; Hopkins et al., 2013), *F. tularensis* OMP subunit vaccines (Mulligan et al., 2017), *Y. pestis* F1/V antigens (Goldberg et al., 2021)	Reactogenicity (LVS), serotype diversity (BoNT)
Antibiotics and antitoxins	Treatment of active infection	Ciprofloxacin (>90% efficacy if ≤ 42h post-plague exposure (Campbell et al., 2020)), raxibacumab (anti-PA MAb), camelid sdAbs (anti-BoNT)	Delayed administration reduces efficacy, toxin resistance

### 6.3 Global collaboration

#### 6.3.1 Strengthening biosecurity frameworks (e.g., WHO’s epidemic intelligence)

Biosecurity frameworks are critical for mitigating risks posed by infectious bacteria as BW agents, yet disparities persist in their global implementation. Studies highlight significant gaps in policy adoption and operationalization, particularly in low-resource settings. For instance, Mutua et al. (2022) identified systemic weaknesses in Kenya’s livestock biosecurity practices, emphasizing the disconnect between national policies and on-ground compliance. Similarly, Standley et al. (2015) demonstrated how international programs like the U.S. Cooperative Biological Engagement strengthened laboratory capacities and disease surveillance in partner countries, underscoring the importance of collaborative funding and training to bridge implementation gaps. These findings stress the need for harmonized, context-specific frameworks that integrate local stakeholder engagement and resource allocation to ensure equitable biosecurity adoption.

Advancements in surveillance systems and ethical governance are pivotal to modern biosecurity strategies. The WHO’s Epidemic Intelligence framework exemplifies a proactive approach, combining real-time data sharing and cross-border collaboration to detect outbreaks early. Brizee et al. (2019) reinforced this by detailing Uganda’s national pathogen inventory system, which enhanced biosafety and biosecurity compliance through centralized monitoring. Concurrently, Xue et al. (2021) advocated for legally binding ethical codes to prevent misuse of dual-use research, aligning with Minehata et al. (2013), who emphasized biosecurity education as a cornerstone for fostering responsible scientific practices. These studies collectively argue that integrating surveillance technologies with ethical governance such as the “One Biosecurity” model proposed by Hulme et al. (2023) can address interconnected human, animal, and environmental health threats while curbing deliberate biological risks.

Effective biosecurity also hinges on robust risk assessment and interdisciplinary coordination. Miller et al. (2017) developed quantitative models to evaluate transboundary disease transmission risks, providing a template for evidence-based biosecurity planning. Kemp et al. (2021) further identified priority research areas, including pathogen prioritization and climate-resilient surveillance, to inform policy. Meanwhile, Khan et al. (2024) highlighted the role of national public health institutes in the Eastern Mediterranean region in coordinating outbreak responses, advocating for institutional capacity-building as a defense strategy. Empirical analyses, such as Filippitzi et al. (2018), quantified the efficacy of biosecurity measures in reducing bacterial transmission, validating their necessity in both agricultural and clinical settings. Together, these findings underscore that strengthening biosecurity frameworks requires a dual focus on innovation (e.g., AI-driven surveillance) and systemic integration of legal, educational, and interdisciplinary tools to counter evolving biological threats. Global frameworks remain critical.

Underpinning all biosecurity frameworks is a crucial discussion of ethics. There are compelling ethical and humanitarian reasons why biological weapons are universally banned under international law, governed primarily by the Biological Weapons Convention. To fortify global defense, policies must incorporate recommendations to strengthen such international treaties, enhance rigorous and transparent inspections of relevant high-containment laboratories, and implement stricter global controls on pathogen access. Furthermore, from a strategic standpoint, the feasibility of deploying biological weapons carries an exceptionally high risk of unintended consequences. The unpredictable epidemiological spread of these agents means deliberate releases carry a severe risk of ‘backfire’ effects, potentially devastating the perpetrators’ own populations or spilling over into unintended, non-combatant civilian populations. Table 5 outlines defense challenges and mitigation strategies.

**TABLE 5:** Key challenges and future defense priorities.

Domain	Challenges	Strategies for mitigation	Post-incident and environmental challenges
Pathogen evolution	Genetic plasticity (e.g., *B. anthracis* clarithromycin resistance (Maxson et al., 2024)), synthetic biology-enabled virulence (Esvelt and Gemmell, 2017; DiEuliis and Giordano, 2017; Gootenberg et al., 2017)	CRISPR surveillance of engineered strains (Moses et al., 2021), multi-epitope vaccines (Jaiswal et al., 2014)	-
Detection gaps	Delayed diagnosis (non-specific symptoms), resource-limited settings	Deployment of field-portable PCR/CRISPR tools (Molsa et al., 2015; Bentahir et al., 2018), AI-driven outbreak prediction (Rainisch et al., 2017)]	-
Environmental remediation	Spore persistence (e.g., *B. anthracis*), wide-area contamination, limited sporicidal agents, hazardous waste management (Plachá et al., 2014; Wood et al., 2021; Franco and Bouri, 2010; Canter, 2005)	EPA-registered sporicides (ClO_2_, H_2_O_2_ vapor); clay-QAC composites; development of RV-PCR for clearance (Plachá et al., 2014; Wood et al., 2021; Kane et al., 2009; Kane et al., 2013; Brisson et al., 2025)	Defining the extent of contamination, scalable decontamination, safe disposal of contaminated materials
Global biosecurity	Disparate infrastructure, dual-use research ethics (Fouet and Mock, 2006; Bakanidze et al., 2010)	WHO Epidemic Intelligence integration (Brizee et al., 2019), “One Biosecurity” frameworks (Hulme et al., 2023)	-
Therapeutic durability	Host-directed toxicity (e.g., anthrax hepatic failure (Liu et al., 2013; Altunkaynak and Ozbek, 2015; Grinberg et al., 2001)), antibiotic resistance	Combinatorial therapies (e.g., toxin inhibitors + antibiotics (Scorpio et al., 2007)), PROTACs targeting BoNT (Tsai et al., 2024)	-

### 6.4 Managing public health emergencies in biological attacks: integrating the CBRNE framework

While advancements in diagnostics, vaccines, and therapeutics (Sections 6.1–6.3) are crucial pillars of biodefense, managing the immediate aftermath of a deliberate biological release demands a highly coordinated, multi-agency operational response. Integrating the established Chemical, Biological, Radiological, Nuclear, and Explosive (CBRNE) incident management framework provides a robust structure for orchestrating this complex response, specifically tailored to the insidious and potentially widespread nature of bacterial bioweapons like *B. anthracis*, *Y. pestis*, and *F. tularensis* (Jernigan et al., 2001; Inglesby et al., 2002; Bower et al., 2023; Cieslak et al., 2018). This framework emphasizes seamless interoperability between public health agencies (e.g., CDC, local health departments), law enforcement (e.g., FBI, local police), emergency management (e.g., FEMA, EPA), and the medical community, acknowledging that a biological attack is simultaneously a criminal act, a public health catastrophe, and an environmental contamination event.

#### 6.4.1 Integrating the CBRNE framework for biological incidents

The CBRNE framework provides a universal language and operational structure for responding to high-consequence events involving hazardous agents (Cieslak et al., 2018; Xue et al., 2021). For biological incidents, it necessitates specialized adaptation focusing on pathogen characteristics (e.g., infectivity, incubation period, environmental persistence) and the critical need for rapid epidemiological investigation concurrent with forensic evidence collection. Key principles include unified command, integrated communications, resource management, and clear delineation of responsibilities across the response lifecycle: from initial detection and identification, through surveillance and situational awareness and containment, to communication and public information and MCM distribution (Bower et al., 2023; Xue et al., 2021). The covert nature of many biological attacks, potentially revealed only by unusual disease clusters, adds complexity compared to overt CBRNE events, demanding heightened vigilance in syndromic surveillance and rapid laboratory confirmation (Section 6.1.1).

#### 6.4.2 Phase I: detection, identification, and initial response

The initial phase hinges on rapid detection and definitive identification of the biological agent, triggering the CBRNE response cascade. Detection relies heavily on robust surveillance systems (clinical, syndromic, and environmental), where emergency departments and primary care providers serve as frontline sensors recognizing unusual symptom clusters such as flu-like illness progressing rapidly to severe respiratory distress (suggesting pneumonic plague) or clusters of severe cutaneous eschars (indicating cutaneous anthrax) (Molsa et al., 2015; Matero et al., 2011; Vieira et al., 2017). Environmental monitoring networks (e.g., BioWatch) may provide early aerosol release warnings (Ho and Duncan, 2005; Rainisch et al., 2017), while citizen reports of suspicious materials necessitate rapid field screening using lateral flow assays or portable PCR (Molsa et al., 2015; Sampath et al., 2012; Saxena et al., 2019). Identification requires swift laboratory confirmation through advanced techniques (multiplex PCR/ESI-MS, CRISPR-based assays, genomic sequencing) in designated Laboratory Response Network facilities for strain typing and resistance profiling (Molsa et al., 2015; Du et al., 2022; Gunnell et al., 2012; Pittman et al., 2002), coordinated with law enforcement for forensic collection. The initial response involves first responders implementing PPE protocols, establishing scene security, isolating exposed individuals, and activating Incident Command Systems and Emergency Operations Centers, with immediate information sharing between public health and law enforcement agencies guiding dual epidemiological and criminal investigations (Jernigan et al., 2001; Bower et al., 2023).

#### 6.4.3 Phase II: surveillance, situational awareness, and containment

Following agent identification, efforts shift to determining incident scope and preventing spread through enhanced surveillance and containment. Intensive case finding and contact tracing, informed by pathogen transmission dynamics (Table 3), employ Geographic Information Systems for spatial mapping and syndromic surveillance to monitor secondary transmission particularly critical for contagious agents like pneumonic plague (Kilgore et al., 2024; Senghore et al., 2023; Vieira et al., 2017). Containment measures include mandatory isolation/quarantine for high-risk scenarios (e.g., pneumonic plague contacts) (Inglesby et al., 2002; Kilgore et al., 2024; Bower et al., 2023), strict healthcare infection controls (respiratory isolation, PPE), and targeted environmental decontamination. For spore-forming agents like *B. anthracis*, EPA-guided remediation ranges from surface disinfection (e.g., sporicides like chlorine dioxide, hydrogen peroxide vapor) to large-scale cleanup (Plachá et al., 2014; Wood et al., 2021), while zoonotic threats require vector/reservoir control (flea control/rodent management for plague; tick/water source control for tularemia) (Eads et al., 2024; Poché et al., 2020). Forensic investigations proceed concurrently, balancing evidence collection with public health priorities.

#### 6.4.4 Phase III: communication, public information, and MCM deployment

Effective crisis management requires clear communication and efficient MCM distribution. Unified command delivers consistent, threat-tailored messaging through multiple channels to manage public fear, prevent misinformation, and ensure compliance with directives (e.g., shelter-in-place, evacuation, prophylaxis seeking) (Jernigan et al., 2001; Ralston et al., 2019), while engaging community leaders. MCM deployment leverages pre-established plans like the Strategic National Stockpile and Points of Dispensing. PEP (e.g., ciprofloxacin/doxycycline for anthrax/plague exposure; possibly post-exposure vaccination like AV7909 if available) targets asymptomatic individuals (Savransky et al., 2017; Bower et al., 2023; Shearer et al., 2021; Russell et al., 1996; Hewitt et al., 2020), while urgent PEP initiates mass dispensing for exposed populations (Inglesby et al., 2002; Shearer et al., 2021; Russell et al., 1996; Hewitt et al., 2020). Symptomatic patients require surge healthcare capacity (ICUs, ventilators for botulism/plague) and rapid access to specific therapeutics (e.g., antitoxins like raxibacumab for anthrax, equine antitoxin for botulism, antibiotics for plague/tularemia) (Hu et al., 2025; Cassie et al., 2024; Sundeen and Barbieri, 2017; Devera et al., 2015). Challenges include MCM triage during shortages, Points of Dispensing crowd management, cold-chain maintenance, and special-needs accommodation mitigated through surveillance integration. Though antibiotics remain primary PEP, ring vaccination may supplement plague outbreak control. The chaotic aftermath of a biological attack demands resilience and adaptability within this CBRNE framework. Continuous evaluation and refinement of national and international response plans, regular multi-agency exercises simulating Category A bacterial agent scenarios, and sustained investment in public health infrastructure and surge capacity are indispensable for mitigating the potentially catastrophic impact of these enduring biothreats (Jernigan et al., 2001; Bower et al., 2023; Cieslak et al., 2018; Xue et al., 2021; Miller et al., 2017).

#### 6.4.5 Phase IV: post-incident characterization and environmental remediation

Following the containment of the immediate public health threat, a prolonged and resource-intensive phase of environmental recovery begins, particularly for agents with high environmental persistence like *B. anthracis* spores (Manchee et al., 1981; Wood et al., 2021). This phase, often termed consequence management, involves systematic characterization of the contaminated area, decontamination, and management of resulting waste streams. The 2001 anthrax attacks in the U.S. starkly illustrated these challenges, where decontamination of postal facilities and office buildings took months and cost hundreds of millions of dollars, revealing significant gaps in protocols for large-scale remediation (Franco and Bouri, 2010; Canter, 2005). A key lesson was the critical importance of robust response and recovery capabilities to minimize the residual risk of latent infections from persistent environmental contamination.

The first step is defining the extent of contamination through aggressive environmental sampling. This involves using wet wipes, vacuum samples, and air samplers to map the dispersion of the agent. To distinguish between a persistent threat and non-viable remnants, culture-based methods are the gold standard but can take 24–48 h. RV-PCR, which couples a short culture step with automated DNA extraction and pathogen-specific PCR, has been developed to provide high-throughput, culture-confirmed detection of viable spores, significantly accelerating clearance assessments (Kane et al., 2009; Kane et al., 2013; Brisson et al., 2025).

Decontamination strategies must be tailored to the agent, the scale of contamination, and the affected material (e.g., buildings, soil, water systems). For indoor environments contaminated with *B. anthracis* spores, fumigation with sporicidal agents like chlorine dioxide or vaporized hydrogen peroxide is often required (Wood et al., 2021). However, these treatments are complex, require sealing the building, and can cause material corrosion. Few antimicrobial pesticides are approved by the EPA for this purpose, and their application often requires specialized vendors, complicating a rapid response (Wood et al., 2021; Scorpio et al., 2007). For outdoor wide-area contamination a worst-case scenario remediation becomes vastly more complex. Lessons from the deliberate contamination of Gruinard Island, which remained uninhabitable for decades, underscore the extreme persistence of spores and the monumental effort required for decontamination, which ultimately involved spraying with formaldehyde and seawater (Manchee et al., 1981). Research into novel decontaminants, such as clay-based composites functionalized with quaternary ammonium salts, shows promise for neutralizing spores on infrastructure surfaces more efficiently (Plachá et al., 2014).

A critical and often underestimated challenge is the management of contaminated waste generated during the response and remediation. This includes contaminated PPE, clinical waste from infected individuals, building materials (e.g., drywall, carpet), and water used in decontamination processes. This waste is often categorized as hazardous and requires secure packaging, transportation, and treatment, typically through incineration at permitted facilities. The logistics of managing thousands of tons of contaminated waste during a large-scale incident pose a massive operational and regulatory challenge that must be integrated into pre-incident planning (Franco and Bouri, 2010). A comprehensive biodefense strategy must look beyond MCMs to include robust, pre-planned protocols for environmental characterization, scalable decontamination technologies, and safe waste management to enable recovery, minimize residual risk, and restore public confidence following a biological incident.

## 7 Conclusion

Infectious bacteria remain central to contemporary biological warfare concerns because they unite efficient transmission routes, potent virulence mechanisms, and, in some cases, extreme environmental persistence. Across *B. anthracis*, *Y. pestis*, *F. tularensis*, *Brucella* spp., and *C. botulinum*, a shared pathogenic logic emerges rapid host entry, early suppression or evasion of innate immunity, and abrupt progression to systemic disease through toxins or dysregulated inflammation. These biological features interact with epidemiological drivers such as incubation period, person-to-person transmissibility, population density, and mobility to determine whether an intentional release results in localized illness or large-scale crisis. Synthetic biology, genomic plasticity, and emerging antimicrobial resistance further compress the margin for error in detection and response.

Effective defense against bacterial biowarfare requires layered, mutually reinforcing strategies rather than reliance on any single countermeasure. Advances in PCR- and CRISPR-based diagnostics, viability-focused environmental assays, and genomic surveillance can shorten time to recognition and forensic attribution. Vaccines such as AVA and AV7909, antibiotics and antitoxins for plague and botulism, and improved decontamination approaches for persistent spores provide critical clinical and environmental tools, but their impact depends on timely deployment within a coordinated CBRNE framework. Robust incident management from early case detection and contact tracing to large-scale medical countermeasure distribution, surge critical care, and long-term remediation must be fully integrated with law enforcement and environmental agencies.

Looking forward, strengthening global biosecurity will hinge on closing capacity gaps in low-resource settings, harmonizing surveillance and data sharing, and embedding ethical governance around dual-use research. Priorities include broad-spectrum and multi-epitope vaccines, host-directed and toxin-targeted therapies, scalable decontamination technologies, and AI-enabled outbreak prediction. Investments in these areas will not only mitigate the consequences of deliberate bacterial attacks but also enhance preparedness for naturally emerging and re-emerging bacterial threats, reinforcing global health security in an era of accelerating biological risk.
